# Evaluation of the Birmingham IBS symptom questionnaire

**DOI:** 10.1186/1471-230X-8-30

**Published:** 2008-07-23

**Authors:** Andrea K Roalfe, Lesley M Roberts, Sue Wilson

**Affiliations:** 1 Department of Primary Care and General Practice, Division of Primary Care, Public and Occupational Health, University of Birmingham, Edgbaston, Birmingham, B15 2TT, UK

## Abstract

**Background:**

Irritable Bowel Syndrome (IBS) is a chronic/common condition that causes a significant effect on the individual (reduced quality of life), society (time lost off work) and health services. Comparison of studies evaluating the management of IBS has been hindered by the lack of a widely adopted validated symptom score. The aim of this study was to develop and validate a disease specific score to measure the symptoms of patients with IBS.

**Methods:**

A self-administered 14-item symptom questionnaire (based on Rome II criteria) was mailed to 533 persons included in a prevalence study of IBS. The reliability of each underlying dimension identified was measured by Cronbach's α. Validity was assessed by comparing symptom scores with concurrent IBS specific quality of life (QoL) scores. Reproducibility was measured by the test-retest method and responsiveness measured by effect size.

**Results:**

379 (71%) questionnaires were returned. The underlying dimensions identified were pain, diarrhoea and constipation. Cronbach's α was 0.74 for pain, 0.90 for diarrhoea and 0.79 for constipation. Pain and diarrhoea dimensions had good external validity (r = -0.3 to -0.6), constipation dimension had moderate external validity (r = -0.2 to -0.3). All dimensions were reproducible (ICCs 0.75 to 0.81). Effect sizes of 0.27 to 0.53 were calculated for those with a reported improvement in symptoms.

**Conclusion:**

The Birmingham IBS Symptom Questionnaire has been developed and tested. It has been shown to be suitable for self-completion and acceptable to patients. The questionnaire has 3 internal dimensions which have good reliability, external validity and are responsive to a change in health status.

## Background

Irritable Bowel Syndrome (IBS) affects large numbers of the population (prevalence estimates ranging from 12% to 30%) [1-7] and accounts for a significant proportion of the workload of both primary care practitioners and gastroenterologists [8]. Treatment options are wide ranging and include symptomatic prescribing, dietary management and a range of psychotherapeutic and complementary therapies. The breadth of the problem and the range of treatment options make this a potential area for much research activity.

The diversity of symptom types and profiles in IBS means that there is no agreement on appropriate determination of efficacy in trials. Researchers have therefore used a range of techniques and measures, which are largely arbitrary and unvalidated.

Three large systematic reviews completed in the UK, Europe and the USA [9-11] demonstrated large variety in the primary outcome measures adopted by trialists, with many trials reporting individual symptoms, such as pain or urgency or more arbitrary outcomes such as percentage of unformed stools. A review of randomised controlled trials of pharmacological treatments [12], noted that symptom data were most commonly collected by daily diary (56% of trials), or during consultation. Only 4/70 included trials reported use of a symptom questionnaire and only 2 of these reported validation of the chosen measure [13,14].

Selection of a suitable outcome measure is further complicated by the fluctuating nature of IBS. It has been suggested [15], that due to the variance of symptoms over time, clinical trials in this area should have a minimum duration of 2–3 months. The increase in psychotherapeutic and complementary therapies such as hypnotherapy for the management of IBS, would suggest that follow-up should be even longer, as such therapies may prove to be effective (and cost-effective) over extended periods by dealing with root causes or the learning of self-management techniques. The use of daily diary data collection strategies over long periods may be unfeasible or may increase drop-out rates leading to a biased sample.

The ROME I, II and III trial committees have consistently agreed that the main assessment of outcome in IBS trials should be patient centred [16-18] and over a decade ago Tally et al [17], strongly proposed the need to develop sensitive outcome measures with multiple domains as a priority in this area. A recent move towards global assessment, led to the adoption of 'adequate relief' as a primary endpoint in some trials [19-21] and a literature review of outcome measures identified a dichotomous 'adequate relief' question as the measure of first choice in assessing global symptomatology [22]. The ROME III committee now indicate that 'adequate' or 'satisfactory' relief are the current standards for primary outcome assessment in treatment trials in IBS [18] and this was supported by a recent review of primary endpoints in IBS trials [23]. It is however generally indicated that integrative symptom questionnaires are also acceptable endpoints subject to appropriate validation [18].

There is the risk that exclusive use of dichotomous or 'adequate relief' endpoints in a disease with such differing symptom profiles may result in the failure to identify therapies of benefit to sub-groups of patients or effective on specific symptoms. The GSRS-IBS symptom score [24], based on Rome I criteria has been shown to discriminate by severity and frequency of disease symptoms. However this tool has not been widely used in practice and its generalisability across all age groups has yet to be demonstrated. A recent visual analogue scale for IBS (VAS-IBS) has been developed [25], however this tool was validated on a sample of women only and requires further testing in clinical practice to establish responsiveness.

Therefore the continuing need for a symptom measure suitable for use in a range of clinical and management trials in the area of IBS remains. The principle characteristics of this tool were determined to be 1) patient reported, 2) suitable for repeated testing over extended time periods, 3) acceptable to patients for ongoing completion, and 4) containing multiple dimensions covering relevant symptoms. This paper therefore describes the development and validation of a tool which meets these requirements, is suitable for self-completion and postal return and fulfils the recommendations of the ROME working group regarding validation of a primary outcome measure [16]. Identification of a well validated and increasingly adopted IBS specific quality of life measure [26,27], limited the development of this new tool to a symptom only score which would be used to complement this.

The aim of this study was to develop a symptom score suitable for use in a range of therapeutic trials in IBS and establish its acceptability, reliability, validity and sensitivity to a change in health.

## Methods

### Ethics

Ethical approval for the study, in which this symptom score validation was embedded, was obtained from North and West Birmingham Local Research Ethics Committees before commencement of the project.

### Population

A computer generated random sample (stratified by deprivation quartile) of 8646 patients, aged 18 and over, registered with participating practices, were mailed in a study estimating the prevalence of IBS in the West Midlands [28]. This mailing contained a general health questionnaire which asked about previous diagnosis of IBS and also questions based on the ROME II [29], diagnostic criteria for IBS. Five hundred and thirty three people were identified who met the Rome II criteria (n = 398) or who had an existing diagnosis of IBS with some current symptoms or the need for medication during the last 6 months (n = 135). This population formed the target population for the validation of the IBS-specific symptom score. Patients with other pre-existing GI conditions were excluded.

### The Birmingham IBS symptom score

The Birmingham IBS symptom score comprised a self-completed questionnaire which consisted of 14 questions based on the frequency of IBS related symptoms and was derived from Rome II: Functional intestinal disorders (see Additional file 1). Each question had a standard response scale with symptoms all being measured on a 6-point Likert scale ranging from 0 = *none of the time *to 5 = *all of the time*. Several authors have suggested measuring symptoms with visual analogue scales (VAS) [30,31], however there is no firm evidence of the superiority of VAS over Likert scales [32,33], therefore the latter were adopted as they were felt to be more familiar to patients and also less labour intensive to analyse.

### Validation

The Birmingham IBS symptom score was sent to persons within 1 to 2 working days of their response to the General Health Questionnaire, which identified them as eligible. To examine construct validity, participants were also sent the IBS-QOL [26], a previously validated disease specific quality of life measure. A freepost envelope was supplied for the return of questionnaires. Individuals who did not return a questionnaire within 21 days were re-mailed on one occasion.

Seven days after the return of the Birmingham IBS symptom questionnaire the participants were sent a repeat symptom questionnaire which was identical except for the addition of a single question to provide data on change of disease status. This asked about changes between completing the first and second symptom score (*Since completing the last questionnaire my abdominal and bowel symptoms have improved/got worse/remained the same*). At the time of completing the first questionnaire participants were unaware that they would be asked to repeat the score. Responsiveness to change was assessed by these data and reproducibility was determined by data relating to persons who reported no change in symptoms.

### Analysis

Acceptability was explored by consideration of the overall response rate (percentage of questionnaires returned) and the completeness of each question (proportion of each individual question with a valid response).

The percentages of subjects selecting either the lowest response (floor) or highest response (ceiling) for each question were examined to evaluate floor and ceiling effects, because a large ceiling or floor effect would limit the ability of the questionnaire to detect change over time.

Factor analysis with varimax rotation was carried out to identify underlying dimensions within the questionnaire. The number of dimensions was determined with Scree plots and eigenvalues (> 1). The items within each dimension were confirmed by a cross-validation analysis, whereby questionnaires were randomly allocated into two subgroups of equal size and factor analysis repeated on each subgroup. To allow comparison with other symptom scores, the items within each dimension were summed and transformed to a scale ranging from 0 (no symptoms) to 100 (all symptoms).

The reliability of each identified dimension was assessed by examination of internal consistency and reproducibility. Internal consistency was measured by Cronbach's α coefficient [34]. Reproducibility was assessed using data from the second mailing (test-retest) and measured with the intra-class correlation coefficient (ICC). The ICC was obtained from analysis of variance [35] and identifies how much variation in scores is due to true difference between individuals and how much is attributable to variability in the measurement.

Construct validity, assessing whether dimensions measure what they say they do, was examined by the relationship of persons' symptom dimension scores with their QoL scores. The associations were measured using Spearman's rank correlation coefficients.

The responsiveness of the tool was measured by the effect size [36,37], for reported changes in symptoms between the first and second mailings. Effect size for each dimension and total score was calculated from the mean change in scores between first and second mailing divided by the standard deviation of scores at baseline.

## Results

### Demography

The questionnaire was mailed to 533 persons. The average age was 50 years (range 18–89); 73% were female (mean age 49 years) and 27% male (mean age 54 years). Eighty-eight percent of the sample was white and 59% had left school by age 16. Thirty-nine percent were in full-time employment; 9% worked part-time; 24% retired, 15% unable to work for health reasons and 3% were unemployed.

### Acceptability

379 (71%) of the 533 eligible population returned the initial Birmingham IBS symptom score questionnaire. The age, sex, ethnicity, education and employment status distributions of the responders were similar to the non-responders.

Individual item completion rates were all very high and ranged from 97.6% to 99.7%. No individual item demonstrated a substantial floor or ceiling effect, all extreme responses being selected by less than 80% of respondents (Table 1) and therefore no questions were rejected on these grounds. The items with the highest response to a single category were *leaked or soiled *and *mucus or slime *where 74% and 57% respectively gave the response of *'none of the time'*.

**Table 1:** Question completion rates and maximum response frequencies

Question	Number completed	% completed	Maximum % response at extremes
Pain	377	99.5	6.1
Loose, watery stools	377	99.5	28.7
Diarrhoea	376	99.2	44.4
Hard bowel motions	374	98.7	22.2
Straining	375	98.9	20.0
Constipation	376	99.2	35.6
Pain after eating	378	99.7	20.4
Sleep problem	377	99.5	36.9
Leaked or soiled	375	98.9	73.9
Urgency	377	99.5	21.5
Mucus or slime	370	97.6	56.8
Unfinished bowel movement	375	98.9	7.7
Flatulence	378	99.7	7.9
Back and shoulder pain	370	97.6	46.8

### Underlying Dimensions

Factor analysis with varimax rotation identified 3 underlying dimensions within the score. Three questions (12,13,14) however produced inconsistent factor loadings when compared with a cross-validation analysis. This suggested that these questions were unreliable and they were therefore excluded from further analysis. The 3 dimensions could best be described as relating to constipation (3 items), diarrhoea (5 items) and pain (3 items), explaining 26%, 22% and 20% of the variation respectively (Table 2). An overall IBS symptom score was also calculated by the summation of the final 11 questions.

**Table 2:** Factor coefficients after varimax rotation

Question	Factor1	Factor2	Factor3	Factor description
Hard bowel motions	0.90			***Constipation***
Straining	0.86			
Constipation	0.89			
				
Loose, watery stools		0.59		
Diarrhoea		0.63		
Leaked or soiled		0.76		***Diarrhoea***
Urgency		0.66		
Mucus or slime		0.70		
				
Pain			0.78	
Pain after eating			0.84	***Pain***
Sleep problem			0.65	
				
*Eigenvalue*	*2.8*	*2.4*	*2.2*	

### Internal consistency

Cronbach's α coefficients were 0.79 for constipation, 0.90 for diarrhoea, 0.74 for pain and 0.75 for overall score.

### Reproducibility

329/379 (87%) questionnaires were returned from the repeat mailing to those who returned symptom scores. Seventy-five percent (248) patients reported no change in symptoms over the 7–10 day period since completion of the initial score. The ICCs calculated were 0.78 for constipation, 0.81 for diarrhoea, 0.75 for pain and 0.78 for overall score.

### Validity

A negative association was found between each symptom dimension and the previously validated IBS-QoL dimensions (i.e. quality of life increased as symptoms decreased). Spearman's correlation coefficients between each scale and symptom scores ranged between -0.1 to -0.3 for constipation; -0.3 to -0.5 for diarrhoea; -0.4 and -0.6 for pain. Overall scores ranged from -0.5 to -0.7 (Table 3).

**Table 3:** Construct validity measured by Spearman correlation coefficients

	Symptom dimension			
QoL dimension	Constipation	Diarrhoea	Pain	Overall

Dysphoria	-0.2	-0.5	-0.6	-0.6
Body image	-0.3	-0.4	-0.6	-0.7
Health worry	-0.3	-0.4	-0.5	-0.6
Food avoidance	-0.1	-0.4	-0.5	-0.5
Social reaction	-0.2	-0.5	-0.5	-0.6
Sexual	-0.2	-0.3	-0.4	-0.5
Relationships	-0.2	-0.3	-0.4	-0.5
Overall QoL	-0.2	-0.5	-0.6	-0.7

### Responsiveness

Mean scores increased for those reporting worse symptoms at the second mailing overall and across pain and diarrhoea dimensions. Conversely the mean scores decreased overall and across all 3 dimensions for all those reporting an improvement in symptoms. Small effect sizes were found for those who reported worse symptoms overall and across all dimensions (0.06 to 0.18); a similar degree of responsiveness was found for those with no reported change in symptoms (0.06 to 0.13). Of those with a reported improvement, medium effect sizes (0.46, 0.53) were identified for the pain dimension and overall scores; and small effect sizes (0.27, 0.32) for the constipation and diarrhoea dimensions (Table 4).

**Table 4:** Responsiveness measured by effect size

Dimension	Health status at 7/10 days	Baseline	Change at 7/10 days	Effect size
		Mean (SD)	Mean (SD)	

Pain				
	Worse (n = 30)	38.2 (21.7)	3.8 (21.6)	0.18
	Same (n = 245)	33.3 (19.2)	-2.4 (12.5)	0.13
	Improved (n = 47)	31.0 (22.1)	-10.2 (14.4)	0.46
Constipation				
	Worse (n = 30)	25.8 (22.0)	-1.3 (16.5)	0.06
	Same (n = 242)	35.0 (27.7)	-1.6 (16.4)	0.06
	Improved (n = 48)	32.2 (25.2)	-6.8 (15.9)	0.27
Diarrhoea				
	Worse (n = 29)	31.2 (24.7)	3.9 (10.7)	0.16
	Same (n = 236)	21.1 (16.9)	-1.4 (10.0)	0.08
	Improved (n = 46)	19.4 (17.0)	-5.4 (11.8)	0.32
Overall				
	Worse (n = 29)	31.6 (16.8)	2.3 (10.6)	0.14
	Same (n = 230)	28.2 (13.3)	-1.6 (8.2)	0.12
	Improved (n = 46)	26.1 (14.0)	-7.4 (9.5)	0.53

The final Birmingham IBS symptom questionnaire is shown in the attached file (see Additional file 2).

## Discussion

Unlike other symptom scores validated using referral-based populations, the validation of this questionnaire was carried out on a large representative community based cohort. Our primary aim was to create a patient reported score for repeated use in trials – in this setting patient interpretation of symptoms is not an issue and could be beneficial, allowing patients to self-define symptoms within tight parameters. Translation to a clinical setting will require further testing especially in light of known differences in the way patients and doctors define key symptoms such as constipation [38].

The high response rate and the high completion rates of all questions demonstrate the questionnaire's acceptability and suitability for self-completion. The possibility of a response bias due to 15% of responders not working because of health problems may exist. However the prevalence of a range of conditions such as diabetes (5%), hypertension (31%) and cancer (4%) are similar to UK figures and thus our responders reflect the general morbidity of a community population. Patients with other pre-existing GI conditions were excluded from the study.

Three underlying dimensions were identified within the questionnaire with Cronbach's alpha coefficients of between 0.7 and 0.9. These are consistent with recommendations of internal reliability [34,39,40] and provide evidence that the items within each dimension are measuring the same characteristic.

The stability of the responses was demonstrated by the high intra-class correlation coefficients of between 0.75 and 0.81. Consequently we can be confident that changes measured by this tool will be due to the effect of treatment and not due to measurement errors of the tool.

Increases in pain, diarrhoea and constipation scores were associated with decreasing quality of life confirming construct validity thus providing evidence that they measure what they purport to. As anticipated, the Qol scores correlated more highly with the pain scale [26].

The decrease in all scores, and corresponding small to medium effect sizes following an improvement in symptoms, indicate the tool's sensitivity to detect a change in health. The small effect sizes found for those with worse symptoms are most probably due to a ceiling or regression to the mean effect. In these cases, baseline scores, with the exception of constipation, were from subjects with greater symptoms than an average IBS sufferer and therefore scores were unlikely to increase. These changes were observed after a short duration of time (7–10 days); however differences at 3 months have also been shown. A randomised controlled trial of gut-directed hypnotherapy demonstrated an improvement in the Birmingham IBS pain, diarrhoea and overall symptom scores at 3 months compared to usual management [41]. An ongoing randomised controlled trial of a dietary product will provide further estimates of responsiveness over time to supplement these results.

The questionnaire performed well for both genders with similar results for reliability, validity and reproducibility being found for men and women. However, gender specific effect sizes could not be reliably estimated due to the small sample of males reporting a change in symptoms.

Whilst the instrument meets validation criteria, it is acknowledged that it may not query the full and diverse range of symptoms experienced by IBS sufferers. The inclusion of additional questions, or the opportunity for patient nomination of personal outcomes (as in MYMOP [42]), may further enhance the tool.

## Conclusion

This IBS specific symptom questionnaire has been appropriately developed and tested. It is patient-centred, suitable for self-completion and is acceptable. The score includes multiple dimensions that cover representative and relevant questions. The dimension and summary scores provide reliable and valid outcome measures of IBS symptoms. High levels of reproducibility indicate that the score is suitable for use in therapeutic trials where it is important to establish that measured changes reflect a real change in status, not just variability in measurement. The increase in effect size, with improvement in symptoms confirms the instrument's ability to measure a clinically meaningful change in health. It is particularly important that a symptom questionnaire is responsive to a change in health if it is to be used as an outcome in a clinical trial.

The severity of symptoms experienced by populations recruited to treatment trials should be reported if generalisability is to be established. The lack of an acceptable and validated disease specific symptom score has hampered the comparison of the efficacy of different management strategies or attempts to synthesise the results of existing studies in the area of IBS. We recommend that future studies of IBS utilise the Birmingham IBS symptom score, together with the previously well validated IBS-QoL questionnaire, to facilitate the production of high quality patient centred research and to enable future comparisons between published studies and meta-analysis of data.

## Competing interests

The authors declare that they have no competing interests.

## Authors' contributions

The study was conceived by SW and LMR. AKR contributed to the design of the study, performed the analysis and interpretation of the data, and drafted the manuscript. LMR and SW participated in the design of the study and helped draft the manuscript. All authors have read and approved the final manuscript.

## Pre-publication history

The pre-publication history for this paper can be accessed here:



## Supplementary Material

Additional file 1 Original unvalidated Birmingham IBS symptom questionnaire with 14 questions. Click here for file

Additional file 2 Final validated Birmingham IBS symptom questionnaire with 11 questions. Click here for file
