# Supplementary material for: Evaluation of the Birmingham IBS symptom questionnaire
Source: BMC Gastroenterol. 2008 Jul 23;8:30. doi: 10.1186/1471-230X-8-30 (PMC2496907; doi:10.1186/1471-230X-8-30)
Supplement: Additional file 1 — Original unvalidated Birmingham IBS symptom questionnaire with 14 questions. [file 1471-230X-8-30-S1.pdf]

BIRMINGHAM IBS SYMPTOM QUESTIONNAIRE (unvalidated)

The following questions ask you about your abdominal and bowel symptoms. When we use the word abdomen we mean belly/tummy. Some of the questions ask about passing a stool. By this we mean going to the toilet for a reason other than to urinate (pass water). All of these questions refer to the <u>last 4 weeks</u>.

Please tick one box for each statement.

	All of the time	Most of the time	A good bit of the time	Some of the time	A little of the time	None of the time
1. During the last 4 weeks, how often have you had discomfort or pain in your abdomen?						
2. How often have you been troubled with loose, mushy or watery bowel motions during the last 4 weeks?						
3. How often during the last 4 weeks have you been troubled with diarrhoea?						
4. During the last 4 weeks how often have you been troubled by hard bowel motions?						
5. During the last 4 weeks how often have you felt the need to strain to pass a motion (stool)?						
6. During the last 4 weeks how often have you been troubled by constipation?						
7. During the last 4 weeks how often did you experience pain or discomfort in your abdomen after eating?						
8. How often has you abdominal pain prevented you from sleeping, or woken you during the night during the last 4 weeks?						
9. During the last 4 weeks how often have you leaked or soiled yourself?						
10. How often during the last 4 weeks have you suffered from a feeling of urgency (feeling that you must immediately rush to the toilet to pass a stool)?						
11. How often have you passed mucus or slime in your stools over the last 4 weeks?						
12. During the last 4 weeks how often have you felt that after finishing a bowel movement there was still a stool which needed to be passed?						
13. How often during the last 4 weeks have you experienced troublesome flatulence (passage of wind)?						
14. During the last 4 weeks how often did you find that your abdominal pain spread to your back and shoulders?						
